# Biodegradation of Aflatoxins by Four Novel Environmental Bacterial Isolates: A Comparative Genomic and Molecular Docking-Proof-of-Concept Study

**DOI:** 10.3390/foods15183242

**Published:** 2026-09-14

**Authors:** Rawan Muhammad Shady, Adel Abdelkhalek, Ahmed Abd El Wahed, Arianna Ceruti, Behrouz Alizadeh Savareh, Ahmed S. Fouad

**Affiliations:** 1 Biotechnology Department, Faculty of Science, Cairo University, Giza 12613, Egypt; rawan.mohamed@buc.edu.eg (R.M.S.); ahmedsfouad@sci.cu.edu.eg (A.S.F.); 2 Department of Food Hygiene, Safety and Technology, Faculty of Veterinary Medicine, Badr University in Cairo (BUC), Badr City 11829, Egypt; 3 Department of Food Hygiene and Control, Faculty of Veterinary Medicine, Mansoura University, Mansoura 35516, Egypt; 4 Institute of Animal Hygiene and Veterinary Public Health, Leipzig University, 04103 Leipzig, Germany; arianna.ceruti@uni-leipzig.de (A.C.); behrouz.alizadeh@uni-leipzig.de (B.A.S.); 5 Department of Medicine II, Heart Center Bonn, University Hospital Bonn, 53127 Bonn, Germany

**Keywords:** aflatoxins, biodegradation, oxford nanopore technology, molecular docking, food safety

## Abstract

Aflatoxin contamination remains a critical challenge for food and feed safety, necessitating effective mitigation strategies. This study addresses the challenge of aflatoxin contamination by identifying four novel bacterial candidates capable of degrading four major aflatoxin congeners (B1, B2, G1, and G2). Quantitative HPLC and qualitative LC-HRMS with MS/MS profiling revealed that the isolated bacterial strains achieved high degradation efficiencies of 86–94%, proceeding through distinct demethylation and decarboxylation pathways. Oxford Nanopore whole-genome sequencing identified these potent degraders as *Escherichia coli* EC_2 and *Klebsiella pneumoniae* (KP_4, KP_5, and KP_6). To elucidate the genomic basis of this phenotype, we performed a comparative genomic analysis against two non-degrading *E. coli* strains. Functional annotation revealed complete aromatic degradation modules and intact enzymatic cascades present only in the degrading strains, demonstrating that this biodegradation capability is highly strain specific. Furthermore, molecular docking of key putative enzymes unique to these active cascades demonstrated highly favorable binding affinities (up to −8.70 kcal/mol) with all four aflatoxin congeners, stabilized by robust hydrogen bonding and hydrophobic networks. These findings provide a novel genomic and structural blueprint for aflatoxin biotransformation, highlighting the specific putative candidate enzymes, paaH and mhpB, for future investigations.

## 1. Introduction

Food safety is a cornerstone of public health and economic stability, influencing both human well-being and the sustainability of global trade. As food and feed systems continue to expand across regions and markets, the risk of food contaminants remains a persistent challenge. Among these are aflatoxins, the “silent killers”, which have been recognized since 1960 [1] as a global threat to both human health and the economy [2]. Aflatoxins are toxic secondary metabolites mainly produced by *Aspergillus* species, particularly *A. flavus*, along with other strains such as *A. parasiticus* and *A. nomius*. On a global scale, aflatoxin exposure affects nearly 5 billion people, highlighting their status as one of the most significant and widely distributed mycotoxin groups of public health concern [3].

The International Agency for Research on Cancer (IARC) classifies aflatoxins as carcinogenic groups. In particular, aflatoxin B_1_ (AFB_1_) was classified as a Group 1 carcinogen in 2002 because of its well-established links to hepatocellular carcinoma and acute aflatoxicosis leading to sudden death [4]. Economically, aflatoxin contamination imposes major losses worldwide. In the United States, US$52.1 million to US$1.68 billion in losses were estimated in the corn industry [5]. In developing countries, aflatoxin-related agricultural losses account for 38% of global trade. Trade restrictions further intensify this impact; European Union regulations are projected to reduce food imports from African countries, including Egypt, Nigeria, Senegal, South Africa, Sudan, and Zimbabwe, by 64%, resulting in losses of about US$670 million annually [6].

Thus, aflatoxin means of treatment and removal have grabbed attention in the last few decades. Methods of removal and prevention varied into physical, chemical, and microbiological methods. Physical and chemical mitigation methods suffer from high costs, incomplete toxin removal, nutrient degradation, and the risk of generating toxic by-products [7]. Microbial degradation provides innovative solutions as a more sustainable, green, and targeted strategy [8]. Microbial removal of aflatoxins occurs primarily through two mechanisms: reversible adsorption onto cell-wall components and irreversible enzymatic degradation. Adsorption is frequently reported in lactic acid bacteria (LAB), probiotics, and yeasts. Consortia of *Lactobacillus, Acetobacter*, and *Saccharomyces* have been reported to remove more than 56% of AFB1 [9]. While enzymatic degradation modifies the aflatoxin structure, commonly by opening the lactone ring, producing metabolites with substantially reduced toxicity [10].

Völkl et al. hypothesized that biological degraders are ubiquitous in nature [11], as aflatoxins do not accumulate indefinitely in natural environments, despite their inherent chemical stability. Consequently, environmental niches serve as rich reservoirs for isolating microorganisms. Despite the successful isolation of various environmental microorganisms capable of aflatoxin biodegradation, the precise molecular mechanisms governing these phenotypes remain largely unresolved [12]. Traditional screening strategies often utilize basic physiological stressors, such as treatment with metal ions, heat, or Proteinase K, to offer preliminary confirmation of the enzymatic nature of the degradation process [13]. While these classical biochemical assays are foundational, they often fail to capture the underlying genetic diversity or elucidate the complete, multi-enzyme metabolic pathways involved [12]. Furthermore, much of the existing literature relies on partial marker-gene sequencing for organismal identification, leaving public databases for phenotypically active degraders highly fragmented and lacking complete genomic context.

The advent of high-throughput “omics” technologies offers a powerful approach to resolve the structural diversity and complexity of these biotransformation pathways. Whole-genome sequencing (WGS) has previously succeeded in identifying specific AFB1-degrading enzymes, such as those characterized in *Bacillus licheniformis* BL-010 [14] and *Aspergillus niger* RAF106 [15,16]. Furthermore, long-read sequencing technologies, such as Nanopore sequencing, overcome the limitations of highly fragmented short-read drafts by providing high-contiguity, closed genomic resolutions, and multiplexing multiple isolates within a single run, thereby facilitating robust and streamlined comparative genomic analyses across diverse microbial strains [17]. However, the systematic application of these genomics-guided pipelines to isolate, screen, and safety-profile environmental strains from agricultural niches in the context of aflatoxin biodegradation remains limited.

To address these gaps, this study suggests a high-throughput discovery framework through a multi-tier pipeline that starts with broad phenotype isolation and systematically narrows down to high-affinity, putative candidate enzymes through the following sequential phases: (i) isolating and screening environmental bacterial strains; (ii) differentiating active microbial biodegradation from passive biosorption; (iii) utilizing long-read Oxford Nanopore sequencing to reconstruct complete genomes and identify taxonomic profiles; (iv) mapping conserved degradation pathways and safety screenings for transmissible antibiotic resistance (AMR) genes; (v) identifying biotransformation metabolites using UPLC-HR-MS/MS mass shift profiling and the byproduct detoxification probabilities; and (vi) predicting putative enzyme-ligand binding affinities via molecular docking.

## 2. Materials and Methods

### 2.1. Reagents, Standards, and Media

An aflatoxin mix was purchased from Sigma-Aldrich Standard Solutions (Sigma-Aldrich, St. Louis, MO, USA) with concentrations of AFB1/G1 of 1 µg/mL and AFB2/G2 of 0.3 µg/mL, respectively. In 5% (*v*/*v*) HPLC-grade methanol, 400 ng/mL of B1/G1 and 120 ng/mL of B2/G2 were prepared as stock solutions. Coumarin 1% *w/v* stock solution (Merck-Schuchardt, Hohenbrunn, Germany) was filter-sterilized (0.22 µm) and diluted into minimal salts broth (MSB) media [3 g/L NaNO_3_, 0.5 g/L KCl, 0.5 g/L MgSO_4_·7H_2_O, 0.01 g/L FeSO_4_·7H_2_O, and 15 g/L agar (Hi-Media, Mumbai, India); pH 7.0 adjusted with 1 M NaOH]. Strains investigated in this study were named uniformly to *Escherichia coli* (EC_1, EC_2, EC_3) and *Klebsiella pneumoniae* (KP_4, KP_5, KP_6).

### 2.2. Sample Collection and Preparation

Microbiological samples were collected aseptically from mycotoxin-contaminated sites across Egypt (Cairo, Alexandria, and Damietta) between September and October 2024 to capture diverse microbial populations exposed to aflatoxins. A total of 12 samples were included: 3 cow fecal materials (swabbed with sterile culture swabs in 5 mL sterile saline), 1 soil sample (aseptically scooped into sterile 50 mL Falcon tubes from herbicide/insecticide-exposed Badr University in Cairo, BUC gardens), 2 mold-contaminated cereal feeds (swabbed from BUC animal laboratory storage), 5 raw cow milk samples (aseptically collected in sterile bottles from local farms), and 1 Romy cheese sample (market-sourced). Samples were transported at 4 °C to the laboratory within 4 h and processed immediately under a laminar flow hood (Biosafety Level 2).

### 2.3. Enrichment, Isolation, and Screening of Coumarin-Tolerant Bacteria

To screen and filter candidate aflatoxin-degrading bacteria safely and cost-effectively, coumarin was utilized as the sole carbon source, exploiting its structural role as the lactone ring backbone of aflatoxins [13,18].

A 0.5 g aliquot of each homogenized sample was weighed and suspended in 5 mL sterile 0.85% saline (Oxoid, Basingstoke, UK). Solid matrices (soil, feed, and cheese) were mechanically crushed using sterile pestles prior to suspension. Suspensions were vortexed (30 s), and sterile swabs were dipped to transfer aliquots into 1 mL nutrient broth (NB; Hi-Media, Mumbai, India). Cultures were incubated at 37 °C for 24 h with shaking (150 rpm) to enrich bacterial populations. Uninoculated NB served as sterility control. Enrichment proceeded in 250 mL Erlenmeyer flasks with 50 mL 0.05% coumarin broth (37 °C, 150 rpm, 72 h). Bacterial pellets from 24 h NB cultures were harvested by centrifugation (6000× *g*, 5 min, 4 °C), washed twice in phosphate-buffered saline (PBS; 0.01 M, pH 7.4), and resuspended to OD_600_ ≈ 0.6 and inoculated into the coumarin-MSB. This enrichment cycle was repeated three times to select tolerant strains. Final turbid cultures were serially diluted in PBS (10^−1^ to 10^−6^), spread-plated onto coumarin agar plates (0.1–0.5% *w/v* coumarin), and incubated (37 °C, 48–72 h). Strains demonstrating rapid growth at 0.5% *w/v* coumarin were purified via two successive single colony restreaks. MSB agar without carbon served as the negative control, and Nutrient Agar (NA) served as the positive control.

### 2.4. In Vitro Biodegradation Assays

#### 2.4.1. Spectrophotometric Screening of Coumarin Tolerant Isolates

Candidate isolates were qualitatively screened by UV-Vis spectrophotometry as mentioned by [13]. Pure isolates (100 µL, OD_600_ 0.5) were inoculated into 5 mL 0.5% *w/v* coumarin broth (37 °C, 24 h, 150 rpm). After incubation, samples were centrifuged (6000× *g*, 10 min); Residual coumarin was extracted with equal-volume chloroform (vortex 1 min × 3, phase separation). The residual coumarin was mixed 1:1 with 50% ethanol: water for spectrophotometry analysis. Samples were analyzed and graphed by LabSolutions UV-Vis (v1.11, Shimadzu, Kyoto, Japan) between λ_200–400_. MSB was used as the blank, and Coumarin was the negative control. According to Equation (1), the tolerance percentage was quantified and compared among isolates:

Tolerance % = ((Initial Absorbance − Treatment Absorbance)/Initial Absorbance) × 100(1)

#### 2.4.2. Comparative Evaluation of Viable Bacteria, Cell-Free Supernatant, and Heat-Killed Biomass for the Four Coumarin Tolerant Isolates

To differentiate bio-transformational degradation from physical cell-wall biosorption, three distinct treatments were prepared for the four candidate isolates, The assay was conducted according to [19,20].

##### Viable Bacteria Assay

Active bacterial cultures (OD600 ~ 0.6 in NB, 450 μL) were spiked with 50 μL of the working aflatoxin mix (final concentration: 40 ng/mL of AFB1/AFG1 and 12 ng/mL of AFB2/AFG2) and incubated in the dark at 37 °C and 150 rpm for 72 h. Non-tolerant coumarin isolates (Strains 1 and 3, isolated from raw milk, which failed to grow on coumarin) and uninoculated NB spiked with the aflatoxin mix served as non-tolerant aflatoxin controls and negative standardized controls, respectively.

##### Cell-Free Supernatant (CFS) Assay

To evaluate extracellular enzymatic transformation, active bacterial cultures were centrifuged (12,000× *g*, 5 min), and the resulting supernatant was filter-sterilized through a 0.22 μm syringe membrane filter to completely remove viable cells. The sterile CFS for each isolate was spiked with the working aflatoxin mix to achieve identical concentration kinetics and incubated under the same conditions as the Section Viable Bacteria Assay.

##### Heat-Killed Control (HKC) Assay

To assess passive physical adsorption, bacterial suspensions adjusted to OD600 ~ 0.6 were autoclaved at 121 °C for 15 min to denature cellular enzymes and eliminate viability. The inactivated biomass was harvested by centrifugation, resuspended in sterile NB, spiked with the aflatoxins, and incubated.

Following incubation, all reactions were centrifuged (12,000× *g*, 5 min). The residual aflatoxins were extracted three times with equal volumes of chloroform to be further quantified. The organic fractions were evaporated under nitrogen gas, and residues were reconstituted in the mobile phase for high-performance liquid chromatography (HPLC) analysis.

#### 2.4.3. Time-Course Analysis of Aflatoxin Removal

Dynamic degradation studies were done according to [21]. A representative highly active strain (Isolate 5) was incubated with the aflatoxin mix under the standardized conditions described in Section Viable Bacteria Assay. Samples were collected at designated intervals (0, 6, 12, 18, 24, 48, and 72 h) to evaluate bacterial growth kinetics measured at OD600 simultaneously with residual aflatoxin concentration. Uninoculated spiked media served as the control.

#### 2.4.4. Quantitative HPLC Analysis

HPLC analysis was carried out according to the Association of Official Analytical Chemists utilizing an Agilent 1260 Infinity II LC system (Agilent Technologies, Santa Clara, CA, USA). Chromatographic separation was achieved on an Agilent ZORBAX Eclipse Plus C18 column (4.6 − 250 mm, 5 μm particle size; Agilent Technologies, Santa Clara, CA, USA). The mobile phase was water: methanol: acetonitrile (6:3:1) with a flow rate of 1 mL/min. The injection volume was 20 μL. The FLD was adjusted with an excitation/emission wavelength of 360/450 nm. The column temperature was maintained at 40 °C [22].

The percentage of aflatoxin removal was calculated using the following Formula (2):

Removal % = (1 − (AF peak Area in Treatment/AF peak Area in Control)) × 100(2)

### 2.5. Isolation and Characterization of Bacterial Genomes

A series of biochemical and physiological tests were done following the standard protocols to characterize the bacterial isolates [23]. To conduct a comparative genomic approach highlighting the specific presence of aromatic degradation modules in active isolates versus inactive. Genomic DNA was extracted using the Easy Pure Bacteria Genomic DNA Kit Protocol (TransGen Biotech Co., Ltd., Beijing, China). An overnight pure single colony was incubated in 5 mL NB (37 °C, 150 rpm, 24 h). In a 1.5 mL microcentrifuge tube, 1 mL of the culture was transferred and centrifuged at (12,000× *g*, 1 min). The kit protocol was followed, and the gDNA was visualized for integrity on a 1% agarose gel and quantified using NanoDrop (Quawell v5.4.0; A260/280 1.8–2.0, A260/230 > 2.0). Whole genome sequencing was performed using the Oxford Nanopore sequencing, including the Rapid Barcoding Kit (SQK-RBK114.24; Oxford Nanopore Technologies, Oxford, UK) for fast library preparation, the MinION Mk1B device, and Flow Cell R10 (FLO-MIN114). Qubit dsDNA HS Assay Kit (Invitrogen, Carlsbad, CA, USA, Q32851) was used for quality measurements. In the MinKNOW software (v24.06.16, Oxford Nanopore Technologies), high-accuracy base calling (HAC) was performed with Dorado Base Caller (7.4.14). Raw nanopore reads in fastq format were analyzed using the Epi2me workflow (v2024.1, Oxford Nanopore Technologies). Read quality, length distribution, and total yield were calculated using FastCat (v0.20.0). Taxonomic classification was performed with Kraken2 (v2.1.3), using minimap2 (v2.28) for read mapping and samtools (v1.21) for downstream file processing. BLASTn (v2.14.0) was used for alignment using Python (v3.10.12).

### 2.6. Genome Assembly, Functional Annotation, and Safety Screening

The merged FASTQ file was de novo assembled using Flye (2.9.6) (-nano-raw mode) with the following parameters: threads: 4, iterations: 2, polished with Medaka (v1.7.2). The annotation was completed by Bakta (v1.12.0) & Prokka (v1.2.0) [24] and the AMR genes from CARD (CARD, v3.2.8) to detect safety concerns of strains [25] in the Proksee platform (v1.0.0) [26], followed by functional annotation using Kyoto Encyclopedia of Genes and Genomes (KEGG) data, and the annotated genes were further investigated for their ability to degrade aromatic or xenobiotic compounds according to their metabolic pathways and Modules [27]. KOBAS (http://bioinfo.org/kobas) software accessed on 28 August 2026 was used to perform integrative KEGG enrichment analysis. The genomic data is submitted to NCBI with accession number Bio Project PRJNA1453922.

### 2.7. Identification of Degraded Metabolites Using UPLC-HR-MS/MS

#### 2.7.1. Sample Preparation and Liquid–Liquid Extraction

To identify putative degradation products of aflatoxins, the aflatoxin standard mixture was incubated with bacterial culture-free supernatant according to the parameters described in Section Cell-Free Supernatant (CFS) Assay. An untreated aflatoxin standard mixture incubated under identical conditions without bacterial inoculation served as the negative control.

#### 2.7.2. Liquid Chromatography (UPLC) Parameters

To unravel the chemical fate and identify biotransformation intermediates of aflatoxins via high-resolution Orbitrap/Q-TOF mass spectrometry. Chromatographic separation was performed using a Sciex ExionLC system (Sciex, Framingham, MA, USA). The sample extract (10 µL injection volume) was injected onto an XSelect HSS T3 C18 analytical column (100 Å, 2.5 µm particle size, 2.1 mm × 150 mm; Waters, Milford, MA, USA), protected by an in-line filter disk pre-column (0.5 µm × 3.0 mm; Phenomenex, Torrance, CA, USA). The column temperature was maintained at 40 °C with a constant mobile phase flow rate of 0.3 mL/min. The binary mobile phase consisted of 5 mM ammonium format buffer in water adjusted to pH 3.0 with 0.1% formic acid (Mobile Phase A) and 100% acetonitrile (Mobile Phase B). The gradient elution profile was programmed as follows: 0.0–1.0 min, 5% B; 1.0–21.0 min, linear gradient to 95% B; 21.0–28.0 min, hold at 95% B; 28.0–28.1 min, return to 5% B; 28.1–35.0 min, re-equilibration at 5% B.

#### 2.7.3. High-Resolution Mass Spectrometry (HR-MS/MS) Acquisition

Mass spectrometry was executed on a Sciex TripleTOF 5600+ hybrid quadrupole time-of-flight mass spectrometer (Sciex, Framingham, MA, USA) operating in positive electrospray ionization (ESI+) mode. Prior to acquisition, the mass spectrometer was tuned and calibrated to ensure mass accuracy and system stability. Instrument tuning was performed using two sequential acquisition protocols: Q1 Tuning Scan: Performed via direct syringe infusion at a flow rate of 5 µL/min over a run duration of 5 min (15 cycles; cycle time: 20 s; dwell time: 2 ms) across a mass range of *m*/*z* 50.0000–1000.0000. Ion source parameters were set as follows: Ion Source Gas 1 (GS1): 20 psi; Ion Source Gas 2 (GS2): 15 psi; Curtain Gas (CUR): 25 psi; Declustering Potential (DP): 200/80 V; and Ion Spray Voltage Floating (ISVF): 5500 V, while TOF-MS Tuning Scan: Performed via syringe infusion at 5 µL/min for 1 min (218 cycles; accumulation time: 0.25 s; dwell time: 2 ms) over *m*/*z* 50.0000–1000.0000. ESI source conditions were: GS1: 20 psi; GS2: 15 psi; CUR: 25 psi; DP: 80 V; Collision Energy (CE): 10 eV; and ISVF: 5500 V. For sample analysis, Information Dependent Acquisition (IDA) was deployed to acquire high-resolution MS1 and MS2 data within a single 35 min injection cycle (cycle time: 0.6502 s; 2584 total cycles). TOF-MS1 Survey Scan: Spectra were acquired over a mass range of *m*/*z* 50.0000–1000.0000 utilizing high-resolution TOF-MS mode. Source gas and voltage conditions were: GS1: 45 psi; GS2: 45 psi; CUR: 25 psi; Temperature (TEM): 500 °C; and ISVF: 4500 V. IDA-MS2 Product Ion Scan: Product ions were acquired across *m*/*z* 50.0000–1000.0000. The DP was kept at 80 V, and the collision energy was set to 35 eV with a collision energy spread (CES) of 20 eV. The IDA switch criteria selected the top 15 candidate ions per cycle that exceeded a threshold of 200 counts per second (cps). Isotope exclusion was enabled within a tolerance of 2.0 Da. To prevent redundant fragmentation of dominant ions, former target ions were dynamically excluded for 3 s after 3 repeats. Dynamic background subtraction was enabled, and the mass tolerance for precursor selection was constrained to 10.0 ppm. System control and initial raw data acquisition were managed using Analyst TF software (version 1.1.1, Sciex).

#### 2.7.4. Data Processing and Mass Shift Profiling

RAW MS data files were processed using MS-DIAL software (v4.9, RIKEN, Tokyo, Japan) for peak detection, deconvolution, isotopic matching, and alignment. Compound identification required an MS1 mass tolerance of 20 ppm, an MS2 fragment tolerance of 20 ppm, and a minimum identification score threshold of 70%. To map biotransformation pathways, mass shifts corresponding to known enzymatic detoxifications of the parent aflatoxins were screened: The specific mass shifts applied to the parent aflatoxins included: decarboxylation: loss of CO_2_ (−43.9898 Da), demethylation: loss of CH_2_ (−14.0157 Da), Hydration: Gain of H_2_O (+18.0106 Da), Oxidation: Gain of O (+15.9949 Da). Candidate degradation peaks satisfying these mass shifts were filtered by cross-referencing their presence in the bacterial treatment samples against their absence in the negative control. Peak verification and final structural curation were conducted using PeakView software (version 2.2, Sciex) and MasterView software (version 1.1, Sciex) to evaluate fragment ion matches, mass error limits, and isotope patterns.

### 2.8. In Silico Toxicity and Cytotoxicity Profiling of the Putative Degraded Products

To predict the safety profiles of the identified degradation products compared to parent AFB1, computational toxicology modeling was executed using the ProTox-3.0 webserver (https://tox.charite.de/protox3) accessed on 26 August 2026. For each molecular structure, qualitative toxicity predictions (“Active” or “Inactive”) and quantitative probability scores (0.00 to 1.00) were calculated for endpoints including mutagenicity, mitochondrial toxicity, and cytochrome P450 activation. A probability threshold of >0.50 was used to define active toxic hazards [28].

### 2.9. Molecular Docking

To computationally assess the structural binding potential between aflatoxin congeners and the proposed enzymes retrieved from the pathways of the degrading strains, Four enzymes were chosen for molecular docking: benzoate/toluate 1,2-dioxygenase α-subunit (benA-xylX), 2,3-dihydroxyphenylpropionate 1,2-dioxygenase (mhpB) for aromatic ring cleavage, 3-oxoadipate enol-lactonase (pcaD) in the β-ketoadipate pathway, and 3-hydroxybutyryl-CoA dehydrogenase (paaH) in the phenylacetate pathway. The enzyme 4-hydroxy-2-oxovalerate aldolase (mhpE) from the non-degrading *E. coli* EC_1 was docked as a negative control.

#### 2.9.1. Preparation of the Ligand

Four aflatoxin variants were included as ligands: aflatoxin G1 (PubChem CID:133065469), aflatoxin G2 (PubChem CID: 2724362), aflatoxin B1 (PubChem CID: 186907), and aflatoxin B2 (PubChem CID: 2724360). Ligand structures were prepared from molecular structure files retrieved from PubChem, followed by sanitization, hydrogen addition, three-dimensional conformer generation, and geometry optimization. MMFF94 energy minimization was used to obtain docking suitable conformations [29,30].

#### 2.9.2. Protein Preparation

Five receptor candidates were predicted from WGS data and prepared by AlphaFold 3. (Google DeepMind, London, UK) for docking: (mhpB), (pcaD), (benA-xylX), (paaH), and (mhpE). Receptor structures were prepared by removing non-essential solvent records, assigning polar hydrogens and atomic charges, and converting the structures into docking-compatible PDBQT format, following AutoDockTools- (v1.5.7, Center for Computational Structural Biology, La Jolla, CA, USA) style receptor preparation [31,32].

#### 2.9.3. Prediction of Active Sites

Binding-site regions were assigned using receptor-specific grid boxes derived from predicted or selected pocket regions. The pocket assignment was guided by cavity-based binding-site prediction, consistent with CB-Dock2 (v2.0, Cao Group, Dalian, China) logic, which integrates cavity detection and docking for protein-ligand blind docking [33]. Molecular docking was performed using Quick Vina 2 (v2.1, Center for Bioinformatics, Marburg, Germany), and the best-ranked pose for each receptor-ligand pair was retained. Docking scores were interpreted comparatively rather than as experimental binding constants [34]. The selected docked poses were inspected to identify conventional hydrogen bonds, carbon-hydrogen bonds, hydrophobic interactions, alkyl contacts, pi-alkyl contacts, pi-sigma contacts, pi-anion contacts, and pi-pi stacking contacts. Interaction distances were reported in Angstroms and were used with the supplied 2D and 3D figures to interpret the most relevant receptor-ligand complexes [35].

### 2.10. Statistical Analysis

Statistical analyses were investigated using degradation percentage, calculated in Equation (2) relative to the untreated control. The datasets were evaluated by two-way analysis of variance (ANOVA) for treatment controls. Effect sizes were estimated using partial eta squared (η^2^) to determine the magnitude of each factor’s contribution to the observed variability. A significance level of *p* < 0.05 was used throughout the analysis. All analyses were performed using SPSS v28 (IBM Corp, Armonk, NY, USA).

## 3. Results

### 3.1. Screening and Selection of Environmental Candidates

**Figure 1:**
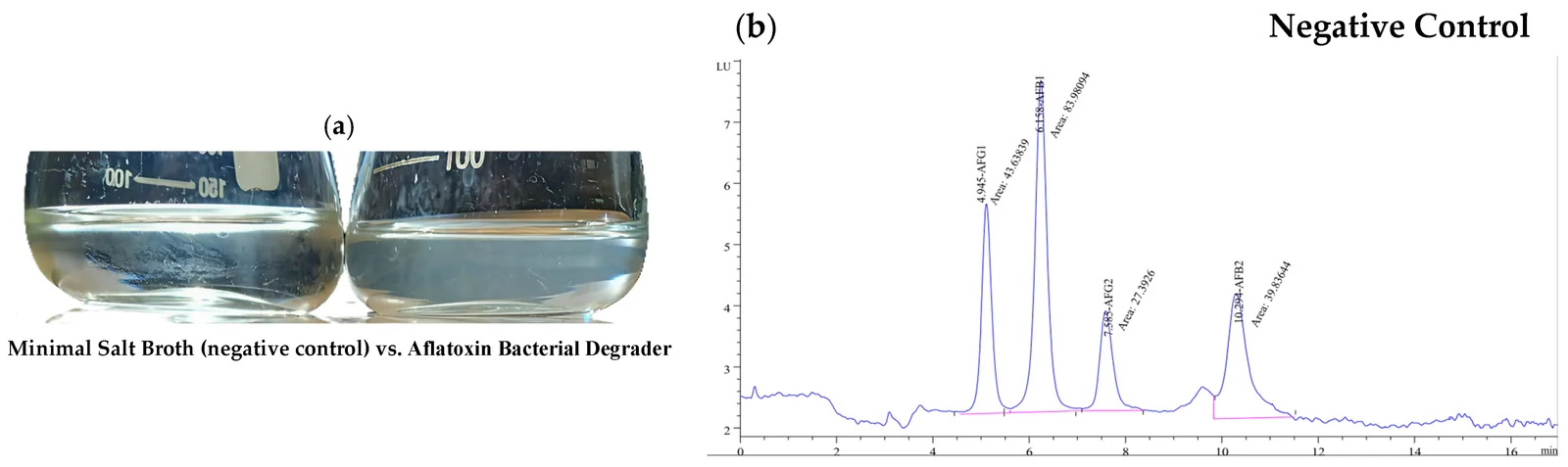

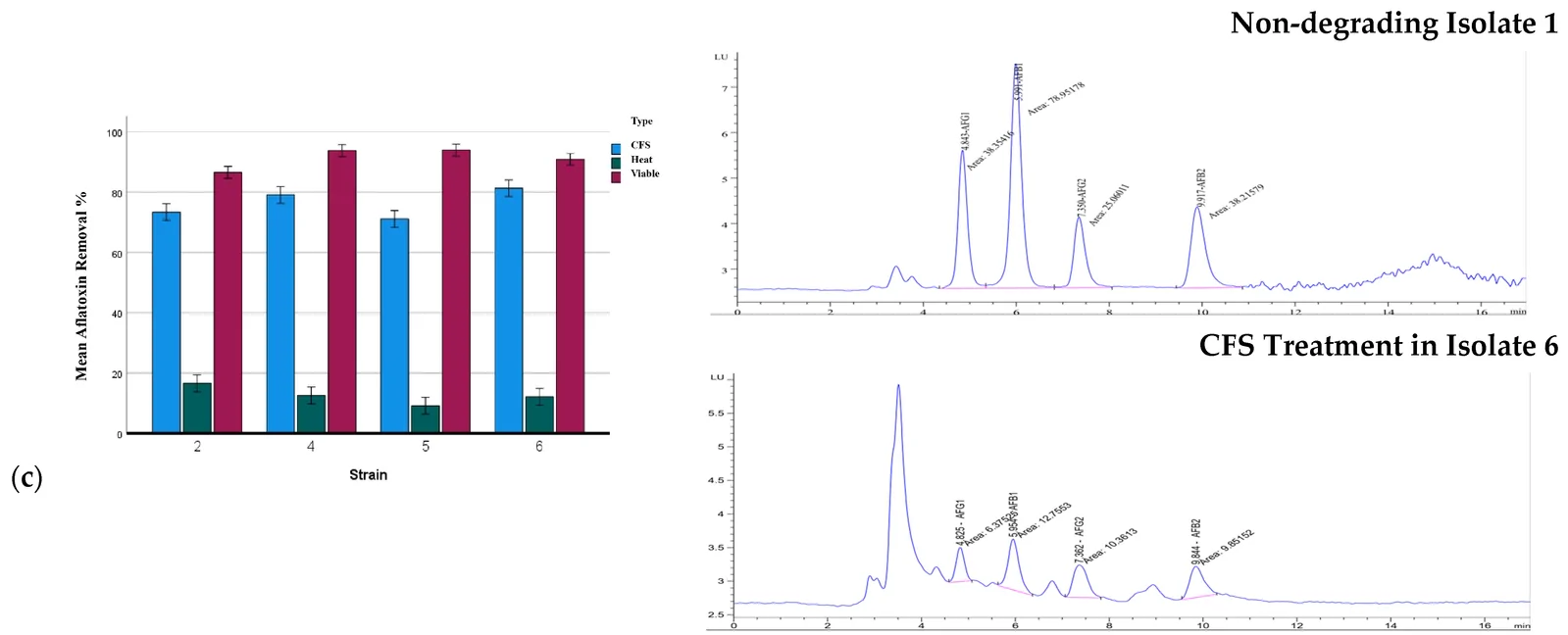
(**a**): Initial screening of aflatoxin bacterial degraders on coumarin broth shows turbidity at (0.5% *w*/*v*), Minimal salt broth as a negative control is clear. (**b**) HPLC chromatograms show the degradation of aflatoxins B1, B2, G1, and G2 of the negative control, non-degrading isolate, and the CFS of degrading isolate respectively. (**c**) Graphical bar representation shows the difference between viable bacteria treatment, cell-free supernatant treatment, and heat-killed biomass across the positive degrading strains; error bars represent standard deviation of the mean (SD).

A total of 12 agricultural, animal, and food-associated samples were screened using coumarin as a structural surrogate for the aflatoxin lactone ring backbone. Selective enrichment on minimal salts broth (MSB) supplemented with 0.5% *w/v* coumarin yielded four highly tolerant bacterial isolates, designated as Strains 2, 4, 5, and 6. These candidate strains demonstrated robust and rapid growth kinetics on coumarin agar plates, using the compound as their sole carbon source, whereas no growth was observed on carbon-free negative control MSB agar plates Figure 1a.

### 3.2. In Vitro Biodegradation Assay

#### 3.2.1. Coumarin Tolerance Screening Profile

Four isolates were selected as promising and strong candidates: 2, 4, 5, and 6. Isolate 6 achieved the highest tolerance rate (96%), followed by isolate 4 (66.90%), isolate 5 (59.50%), and isolate 2 was the least (51.30%). The UV–visible spectra are presented in Figure S1. In contrast, isolates 1 and 3 failed to proliferate in coumarin-supplemented media and were subsequently utilized as non-tolerant reference controls.

#### 3.2.2. Comparative Evaluation of Viable Bacteria, Cell-Free Supernatant, and Heat-Killed Biomass for the Four Coumarin Tolerant Isolates

To differentiate between active biodegradation and physical adsorption by cell wall matrices, three treatment controls were tested: viable bacteria, cell-free supernatants (CFS), and heat-killed biomass (HKC) in Table 1.

As shown in Table 1, viable bacteria treatments consistently yielded the highest removal rates, peaking at (94.02 ± 0.35) for Strain 5. The CFS fractions also demonstrated high efficiency, ranging from (71.04 ± 2.11) (Strain 5) to (81.31 ± 1.52) (Strain 6). In contrast, the HKC preparations showed a significant drop in performance, achieving only minor removal rates between (9.14 ± 3.68) and (16.62 ± 1.72). Two strains (1,3) that did not show any growth on coumarin media were included as non-degrading controls on HPLC. The mean aflatoxin removal (%) for them was 7.72 ± 3.47% and not detected, respectively. Figure 1 displays HPLC chromatograms (b) and graphical bar plot to differentiate the treatment groups and controls (c).

The two-way analysis of variance (ANOVA) evaluated the influence of the microbial strain and the treatment preparation type (viable bacteria, CFS, or HKC) on aflatoxin removal efficiency represented in Table 2.

The two-way ANOVA revealed that the treatment preparation type was the primary driver of variation in aflatoxin removal, accounting for the vast majority of the experimental variance (Partial η^2^ = 0.952, *p* < 0.001). Conversely, differences between the individual strains did not reach statistical significance (*p* = 0.149), despite showing a relatively high partial eta squared value (Partial η^2^ = 0.711). The interaction between the strain and the treatment type (Strain × Type) was also non-significant, demonstrating that the relative performance trend among viable bacteria, CFS, and HKC remained consistent across all four isolates.

#### 3.2.3. Time-Course Analysis of Aflatoxin Removal

**Figure 2:**
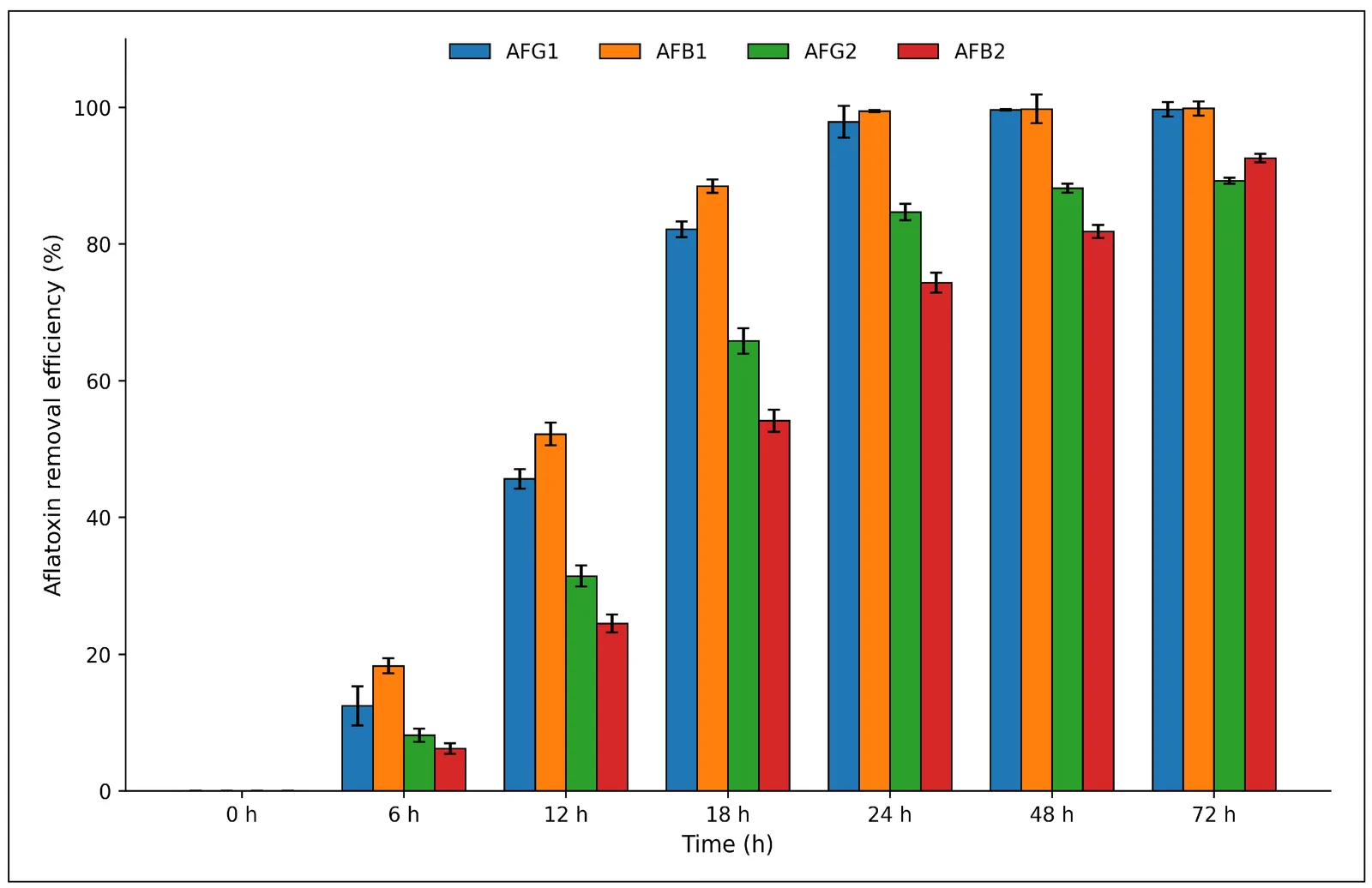
Time-course analysis of aflatoxin removal efficiency (%) by Isolate 5; error bars represent standard deviation (SD) of triplicate experiments.

The removal rate of the AFB1, B2, G1, and G2 was quantitatively monitored over a 72-h period using Isolate 5 as a representative high-efficiency degrader. The removal of all four target toxins commenced during the early exponential phase (6 h to 12 h) and progressed rapidly as the culture approached its growth phase. By 24 h, remarkable biodegradation rates were achieved for AFB1 (99.43 ± 0.15%) and AFG1 (97.88 ± 2.32), while AFG2 (84.67% ± 1.20%) and AFB2 (74.33% ± 1.45) showed moderate degradation. Maximum degradation performance was achieved at 72 h, resulting in near-complete elimination of AFB1 (99.83 ± 1.04%) and AFG_1 (99.70 ± 1.06%), while AFG2 and AFB2 reached high removal rates of (89.25% ± 0.45%) and (92.58% ± 0.62%), respectively, by the end of the 72-h study presented in Table 3 and Figure 2.

### 3.3. Identification and Characterization of the Bacterial Strains

A series of biochemical tests according to Bergey’s manual were performed to initially identify both degrading and non-degrading strains presented in Table 4 and Figure S2.

### 3.4. Comparative Genome Assembly, Annotation, and Biosafety Profiling

**Figure 3:**
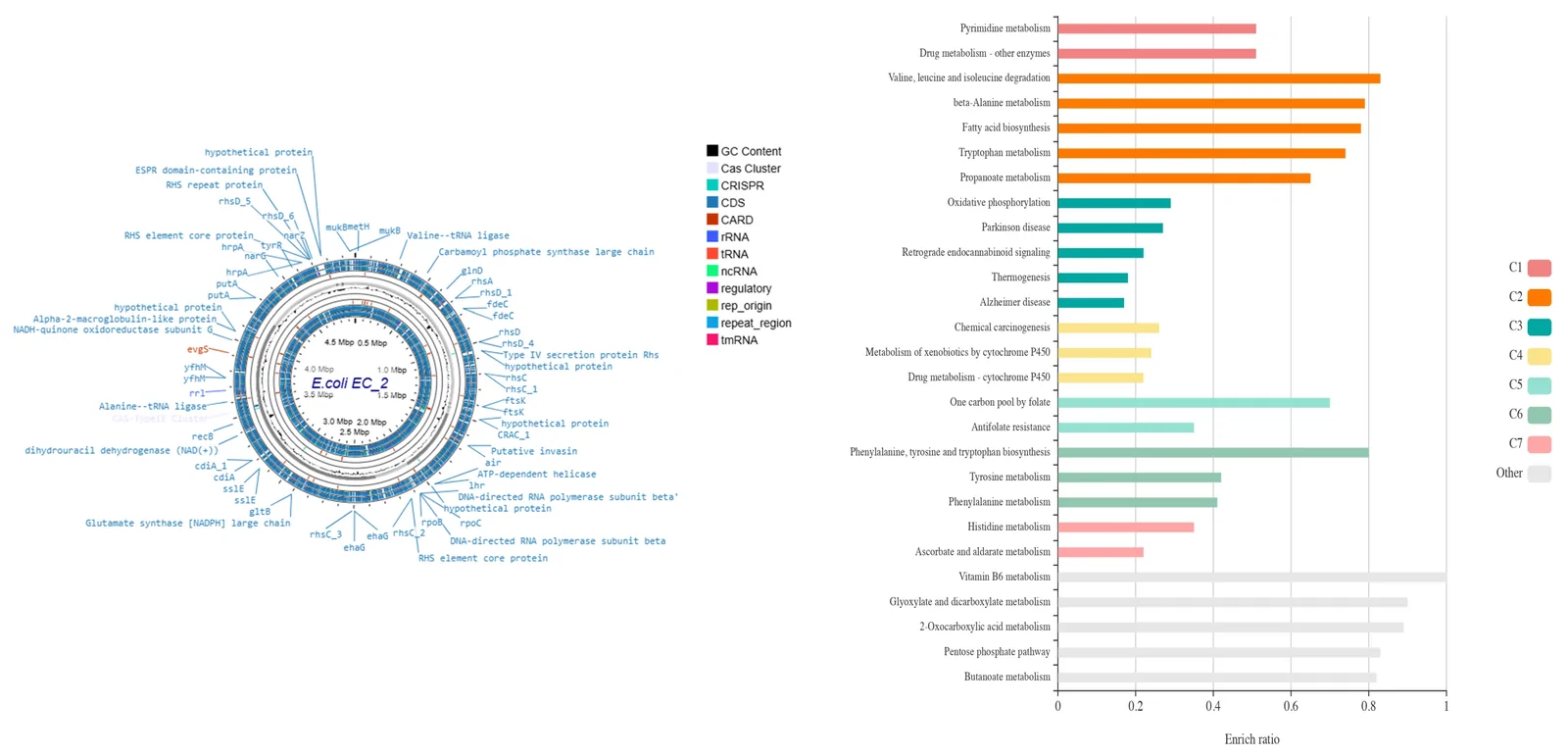

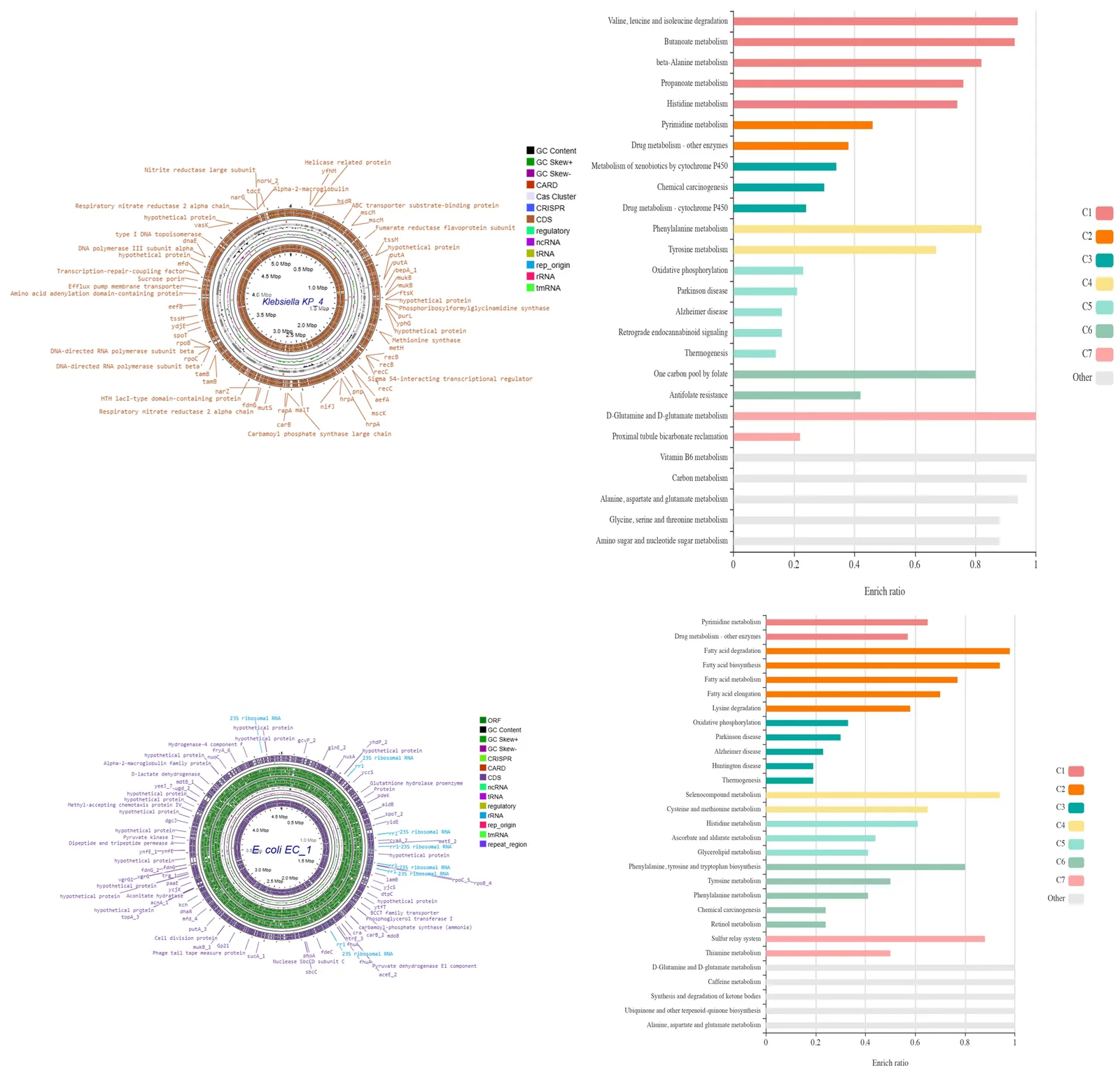
Comparative WGS and the pathway KEGG enrichment analysis of both degrading and non-degrading strains. EC_2 and KP_4 were degrading isolates, while EC_1 was the non-degrading isolate.

Genome assembly statistics provided a solid foundation for subsequent functional annotation. Strains 2, 4, 5, and 6 demonstrated reasonable contiguity despite varying read depths, ranging from 34.7 k reads (strain 4) to 161.4 k reads (strain 6). The *Klebsiella pneumoniae*-like strains (4–6) showed higher GC content (57.1–57.5%) than the *Escherichia coli*-like strains (1–3) at 50.3–50.7%, consistent with their taxonomic assignment. KEGG annotation totals were generally higher in the *Klebsiella pneumoniae* group (3360–3557 proteins) than in the *Escherichia coli* group (2360–3314 proteins), hinting at greater functional potential. The detailed genomic features are found in Table S1 (S1) and Figure 3.

Comparative KEGG pathway reconstruction revealed a pronounced functional difference between xenobiotic-degrading and non-degrading bacterial isolates. *E. coli* EC_2 strain and *Klebsiella pneumoniae* strains (KP_4–KP_6) exhibited complete KEGG modules for Aromatic Xenobiotics degradation, whereas *E. coli* EC_1 and EC_3 lacked these intact pathways. The comparative KEGG modules are detailed in Table S1 (S2). Detailed KEGG module reconstruction and mapping demonstrated that only the degrading isolates maintained intact enzymatic cascades for aromatic xenobiotic breakdown. Complete modules for benzoate degradation (M00551) and catechol *ortho*-cleavage (M00568) were present in *Klebsiella pneumoniae* strains (KP_4, KP_5, and KP_6), each showing full pathway completion (2/2, 7/7, and 4/4 steps, respectively). Phenylacetate degradation (M00878) (7/7) was also complete in all degrading strains EC_2, KP_4-KP_6. All these modules were absent or partial in the non-degrading strains. The presence of a complete set of genes required to initiate, mediate, and terminate each reaction—from benzoic acid conversion to 3-oxoadipate, only in the degrading isolates may explain their observed functionality. Detailed modules and proposed pathways are found in Table S1 (S3).

Genomic screening against the CARD database raised potential biosafety concerns. CARD-identified genes are detailed in Table S1 (S4). Genome analysis revealed the presence of multiple intrinsic multidrug efflux systems in *E. coli* EC_2, including members of the *Acr*, *Mdt*, and *Emr* families. While *Klebsiella pneumoniae* strains (KP_4–KP_6) harbored multiple antibiotic resistance determinants, including SHV-27, a β-lactamase associated with antibiotic inactivation, alongside several efflux pump systems such as oqxA/oqxB, KpnE/F/G, and MdtQ, which are known to contribute to multidrug resistance by reducing intracellular antibiotic accumulation.

### 3.5. Mass Spectrometry Profiling of Putative Biotransformation Metabolites

**Figure 4:**
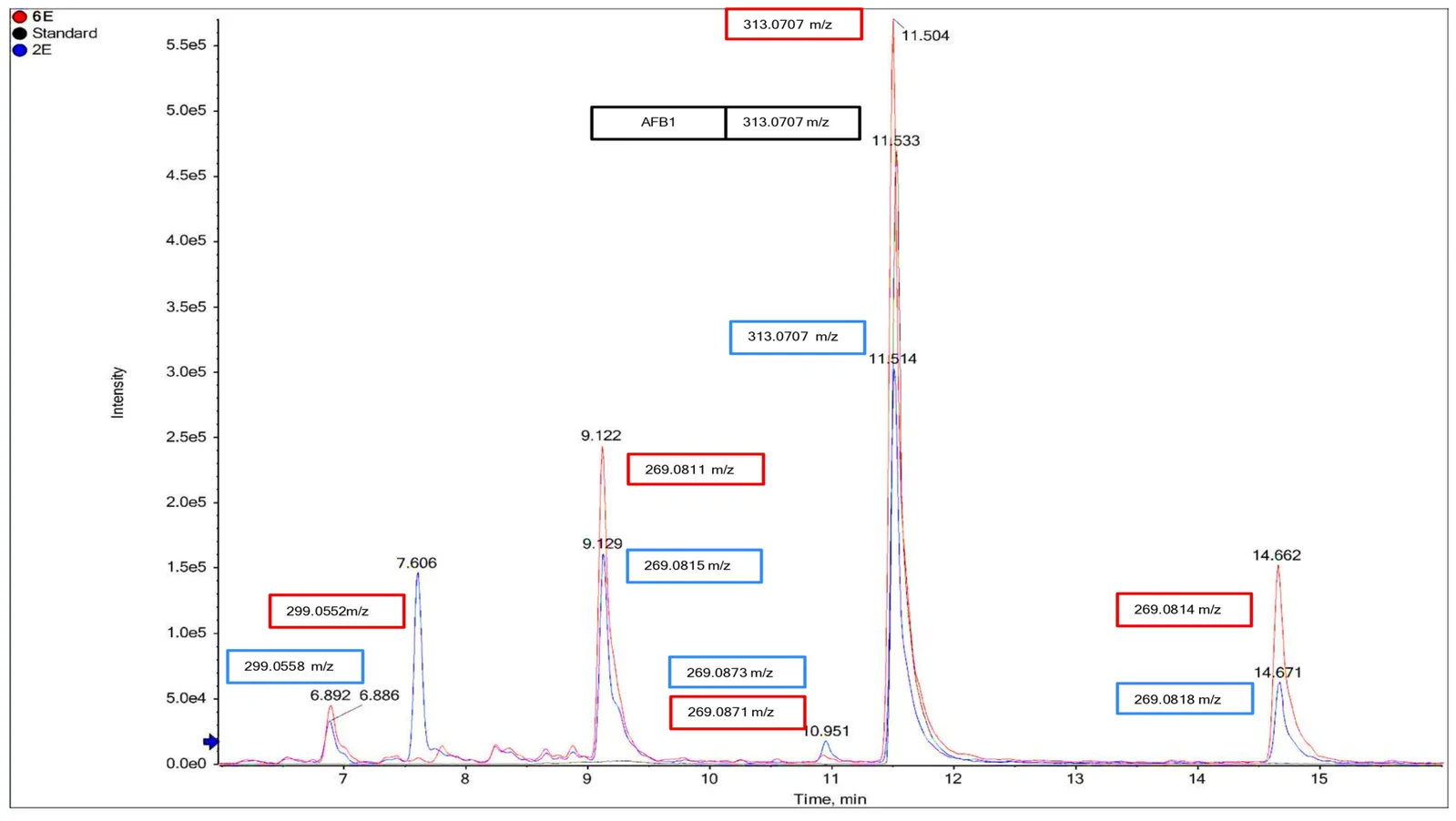
Comparative LC–HRMS profiling of CFS treatments *E. coli* EC_2 and *Klebsiella* KP KP_6 highlighting putative aflatoxin transformation products absent from the aflatoxin standard. Blue boxes indicate *E. coli* EC_2 (2E), red boxes indicate *Klebsiella* KP_6 (6E).

High-resolution Orbitrap/Q-TOF LC-HRMS/MS analyzed the chemical fate of AFB1 following incubation with the CFS of *E. coli* EC_2 and *K. pneumoniae* KP_6. While the parent AFB1 ~ (313.0707 *m*/*z*) with the molecular formula [C_17_H_13_O_6_] + appeared in all groups, with the same RT at ~11.51 min. However, there were newly formed peaks that appeared in the bacterial-treated groups that were not detected in the control presented in Figure 4 and Table 5.

Product P1 (*m*/*z* 299.0558/299.0552) matches the formula [C_16_H_11_O_6_] ^+^ (theoretical *m*/*z* 299.0551). This corresponds to a net loss of a methyl group (CH2, −14.0157 Da) from the methoxy group attached to the benzene ring of the AFB1 coumarin core. Products P2, P3, and P4 all share an observed precursor ion that aligns with the molecular formula [C_16_H_13_O_4_] ^+^ (theoretical *m*/*z* 269.0809). This molecular formula represents a loss of carbon dioxide (CO_2_, −43.9898 Da) from the parent aflatoxin B1. The presence of four distinct chromatographic peaks (ranging from RT 9.117 to 14.671 min) sharing this exact molecular formula suggests the formation of multiple structural isomers around the parent molecule.

### 3.6. In Silico Toxicity and Cytotoxicity Profiling of the Putative Degraded Products

**Figure 5:**
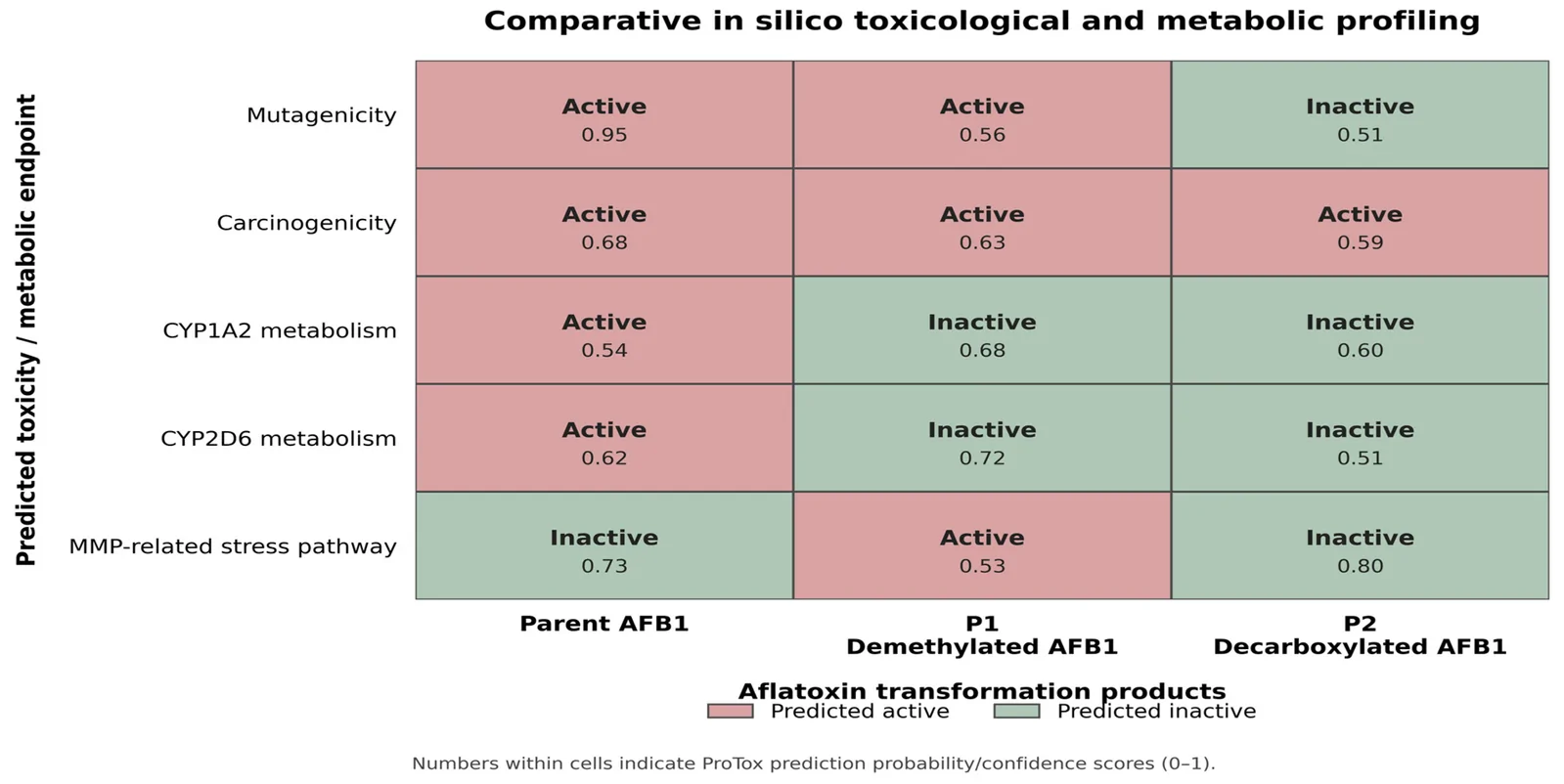
Heat map of silico toxicity and metabolic profiles of Parent AFB1, P1: (demethylated AFB1), P2: (decarboxylated AFB1).

Computational toxicological profiling was performed using the ProTox-3.0 platform to assess the safety of the identified biotransformation products Figure 5. Parent AFB1 was flagged as highly mutagenic (0.95) and carcinogenic (0.68), with a high probability of activation by hepatic cytochrome P450 enzymes (CYP1A2 and CYP2D6). The demethylated intermediate (Product P1) showed reduced mutagenic probability (0.56) and ceased interaction with P450 enzymes, though it showed a transient risk of mitochondrial membrane potential (MMP) disruption (0.53). Significantly, the terminal decarboxylated metabolite (Product P2) was predicted to have a substantially reduced probability of mutagenicity (0.51 probability for inactive status) and lacked any mitochondrial toxicity or hepatic metabolic activation, predicting substantial biological detoxification.

### 3.7. Molecular Docking

In silico molecular docking simulations explored the spatial fit configurations of the putative genomic enzymes against the four target aflatoxin ligands in Table 6.

The docking energy profiles highlighted the 3-hydroxybutyryl-CoA dehydrogenase (paaH) of the phenylacetate pathway as the highest-affinity candidate. It yielded high compatibility scores ranging from (−8.00 kcal/mol) to (−8.7 kcal/mol), with peak affinity exhibited toward AFG2 (−8.70 kcal/mol). The 2,3-dihydroxyphenylpropionate 1,2-dioxygenase (mhpB) served as a strong secondary candidate from (−6.0 to −6.7 kcal/mol), demonstrating the highest affinity toward AFB2 (−6.7 kcal/mol). The negative control enzyme (mhpE), retrieved from the non-degrading *E. coli* EC_1 genome, yielded highly unfavorable positive or marginally negative spatial fit energies, indicating a structural inability to accommodate planar aflatoxin molecules.

**Figure 6:**
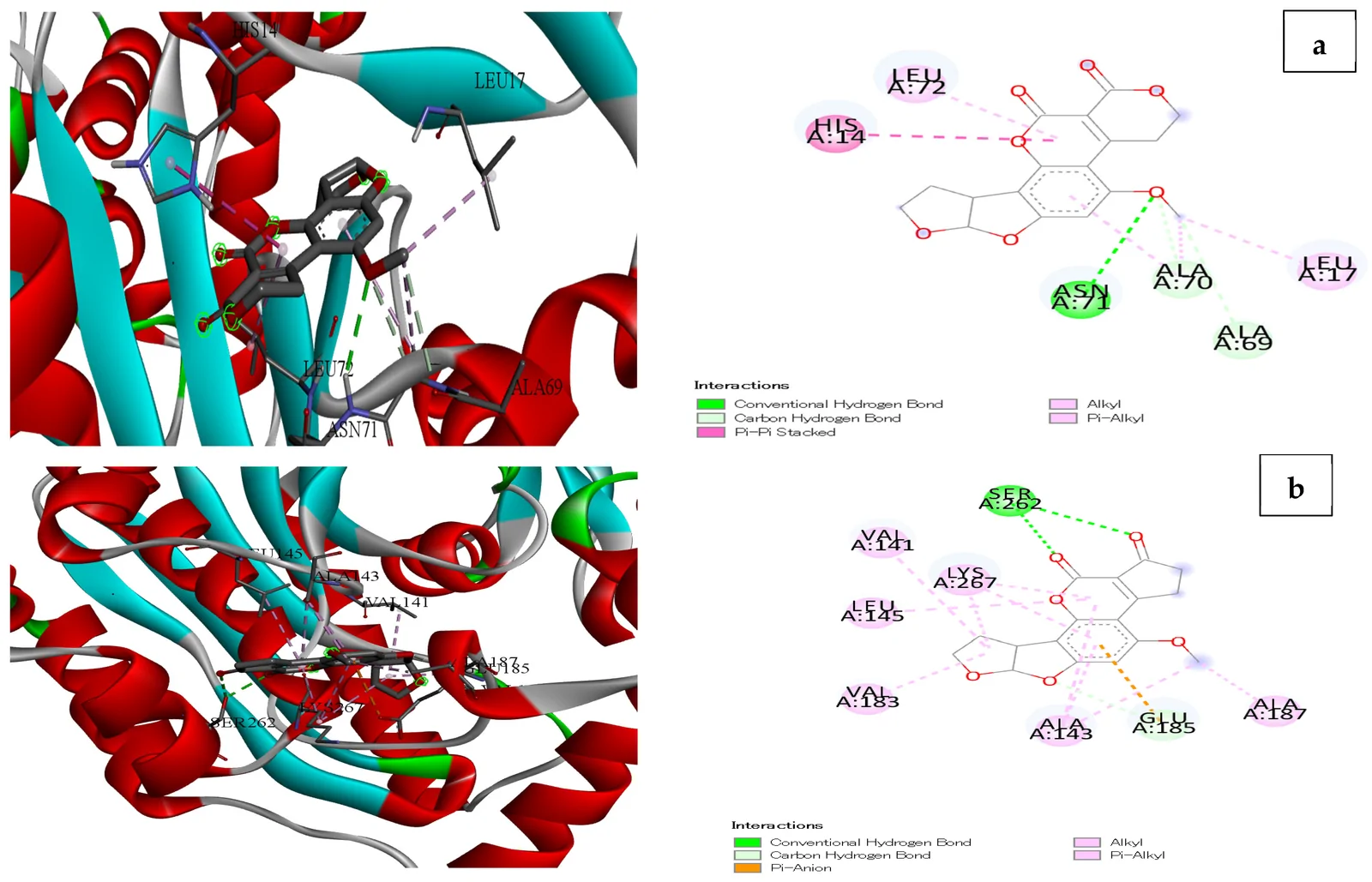
(**a**) Three-dimensional and two-dimensional interaction views of Aflatoxin G2 with paaH. The interaction pattern includes conventional hydrogen bonding, carbon-hydrogen bonding, alkyl contacts, pi-alkyl contacts, and pi-pi stacking features. (**b**) Three-dimensional and two-dimensional interaction views of Aflatoxin B2 with mhpB. The pose is supported by multiple pi-alkyl contacts and alkyl contacts.

Visual analysis represented in Figure 6 of the top-ranked complexes resolved the molecular stabilizing interactions. The paaH–AFG2 complex featured one conventional hydrogen bond (ASN71 at 2.9 Å), two carbon-hydrogen bonds (ALA70 at 3.37 Å and ALA69 at 3.68 Å), and additional hydrophobic packing. While the mhpB–AFB2 complex was stabilized by two conventional hydrogen bonds with (SER262 at 2.92 Å and 2.50 Å), one carbon-hydrogen bond (GLU185 at 3.36 Å), and several hydrophobic interactions, including alkyl and π-alkyl contacts with VAL183, ALA187, LYS267, and ALA143 (distances 4.19–4.40 Å). These mixed stabilizing interactions align with the oxygen-rich and aromatic properties of aflatoxin molecules.

## 4. Discussion

Environmental niches are promising reservoirs for screening and isolating aflatoxin-degrading agents [11]. Thus, selective enrichment strategies are foundational to isolate functional strains. It has been established by Guan et al. [18] that they isolated *Stenotrophomonas maltophilia* 35-3 as a potent agent for AFB1 degradation on coumarin media; the structural backbone of aflatoxins, their screening method yielded zero false positives: 100% of the 25 isolates selected successfully degraded AFB_1_ via the enzymatic pathway. Therefore, this screening method, using coumarin medium as a sole carbon source, utilizing the current study, yielded four potent aflatoxin-degrading isolates (EC_2, KP_4, KP_5, and KP_6).

The four coumarin-tolerant isolates demonstrated robust removal of the four major aflatoxins (B1, B2, G1, and G2), with mean removal efficiencies exceeding 80% for viable bacterial treatments. Misinterpreting temporary physical adsorption as permanent biodegradation can result in the subsequent release of bound, active toxins within the gastrointestinal tract [36]. Therefore, by comparing viable bacterial treatments, cell-free supernatants (CFS), and heat-killed controls (HKC), this study resolved these underlying mechanisms of the four potent strains [36]. The dramatic decrease in aflatoxin removal observed in the heat-treated biomass (HKC) groups (12.62 ± 2.63%) compared to the viable treatments provides key insights that the physiological mechanisms are primarily biological reactions. Because heating denatures proteins and inactivates cellular metabolism, the low, residual activity in the HKC group can be attributed purely to passive physical adsorption [21]. Instead, the high efficacy of the cell-free supernatant (76.25 ± 1.39%) suggests that the removal is driven primarily by active, extracellular enzymes secreted by the bacteria. These came in alignment with studies that identified *E. coli* and *Klebsiella* in their investigations. *E. coli* degrading activity was previously reported by [37], as they demonstrated that *E. coli* CG1061 is a highly effective AFB1-detoxifying strain capable of removing over 90% of AFB1 through a heat-stable, extracellular metabolite. While *Klebsiella* strains were top performers in aflatoxin removals and achieved 71.91% and 64.85%, respectively, as reported by [38] and [39] under extracellular conditions.

Dynamic degradation analysis suggested a distinct, congener-specific degradation hierarchy (AFB1 ≈ AFG1) > AFG2 > AFB2. This can be attributed to their structural differences and degree of saturation [40]. AFB 1 and AFG1 both possess a double bond (C_8=C_9 position) in the terminal difuran ring [40]. This double bond is a highly reactive, electron-rich center that is particularly susceptible to rapid biological cleavage. This structural vulnerability explains why AFB1 and AFG1 underwent rapid elimination. In contrast, AFB2 and AFG2 lack this C_8=C_9 double bond in their terminal furan rings, rendering their structures more saturated and chemically stable. Consequently, they exhibit greater resistance to initial microbial elimination, resulting in a slower, more progressive removal curve over the 72 h period.

By employing long-read Oxford Nanopore sequencing, the limitations of fragmented short-read drafts were bypassed, and the complete genomic architecture of these active strains is resolved [17]. Comparative genomic and functional annotations revealed clear distinctions between degrading and non-degrading isolates. Kyoto Encyclopedia of Genes and Genomes (KEGG) pathway enrichment analysis provides comprehensive insights into the functional genes and metabolic pathways involved in the degradation of aflatoxin. Similar enrichment approaches have previously been employed to characterize the functional genes of plant growth [41,42].

KEGG pathway reconstruction demonstrated a high difference in xenobiotic degradation. Degrading strains 2, 4, 5, and 6 possessed significantly higher counts of genes assigned to Microbial metabolism in diverse environments, Degradation of aromatic compounds, and Benzoate degradation modules than the non-degrading strains EC_1 and EC_ 3.

Detailed module reconstruction further clarified this pattern. Only the degrading isolates maintained complete, intact enzymatic cascades for key steps in aromatic xenobiotic breakdown. Complete modules were identified for benzoate degradation (M00551; 2/2 steps), catechol *ortho*-cleavage (M00568; 4/4 steps) in strains KP_4–KP_6, and phenylacetate degradation (M00878; 7/7 steps) in strains EC_2 and KP_4–KP_6. These pathways were either absent or present only as fragmented, incomplete versions in the non-degrading *E. coli* strains EC_1 and EC_3. The specific gene products (e.g., K05549, K05550, K05783–K05784, K01055, K01856, and others annotated under these modules) encode the full sequence of enzymes required to initiate, catalyze, and terminate conversion of aromatic intermediates to central metabolites such as acetyl-CoA, succinyl-CoA, and 3-oxoadipate. This genomic completeness can support the observed degradation activity in the positive strains and its absence in the negative controls. The present comparative analysis therefore represents a novel contribution: it demonstrates that the presence of these intact KEGG modules in non-LAB Gram-negative bacteria represents a key candidate pathway that could pinpoint their aflatoxin-degrading capability.

Although the investigated *E. coli* strains shared high ANI percentages (98.3–99.2%), they exhibited distinct functional disparities. This highlights that metabolic capabilities are often strain-specific traits acquired in response to environmental stress. This finding comes in line with the Pan-genome analysis done by [16] on 32 related strains, which demonstrated that aflatoxin B_1_ degradation of *Rhodococcus* strains was highly strain-specific, further revealing substantial heterogeneity in metabolic pathways, regulatory networks, and stress-response systems even among closely related isolates. The current study further emphasizes that observed differences arise from specific metabolic modules rather than phylogenetic distance between *E. coli* strains.

The taxonomic identification of *E. coli* and *K. pneumoniae* highlights a critical biosafety challenge in bioremediation: many highly active environmental degraders are opportunistic pathogens. Screening these isolates against the Comprehensive Antibiotic Resistance Database (CARD) revealed an abundance of resistance genes dominated by efflux-mediated mechanisms, alongside genes associated with reduced membrane permeability and antibiotic inactivation (e.g., *SHV* variants). The abundance of multidrug efflux systems (e.g., *mdt*, *acr*, *oqx*) presented in Table S1 (S4) suggests a constitutively active export capacity, which is not limited to antibiotics but extends to structurally diverse chemical compounds [40]. This likely explains the observed tolerance toward coumarin and aflatoxins, where active extrusion may contribute to the apparent tolerance phenotype. However, the presence of these transmissible antimicrobial resistance (AMR) genes makes it unsafe to apply these viable cells or crude CFS directly to food or feed systems, due to the risk of endotoxin contamination and horizontal gene transfer. Fortunately, this limitation will not diminish their value as genetic reservoirs for enzyme mining. Historically, opportunistic pathogens have served as highly valuable templates for identifying industrially useful enzymes. The genus *Nocardia* contains opportunistic pathogens, yet its genomes are heavily mined for novel biocatalysts and secondary metabolites. Similarly, *Serratia marcescens* is a clinical opportunistic pathogen widely used in biotechnology for its diverse enzymatic repertoire [43].

Therefore, the safest and most viable application strategy is to identify the specific genes responsible for degradation and express them in safe, food-grade heterologous hosts (such as *Saccharomyces cerevisiae* or *Bacillus subtilis*).

Ultra-Performance Liquid Chromatography High-Resolution Mass Spectrometry (UPLC-HR-MS/MS) profiling confirmed that the extracellular enzymes in the CFS of *E. coli* EC_2 and *Klebsiella* KP_6 target these structural features: demethylation and decarboxylation in putative degraded products (P2–P4): the loss of CO_2_ (43.99 Da) is a chemical indication of the decarboxylation of the lactone ring [44]. While demethylation in P1 formed C_16_H_10_O_6_: replacing the methyl group with a hydrogen atom converts the methoxy group into a phenolic hydroxyl group, which reduces the electrophilicity of the molecule [44]. Cleaving the lactone ring is essential for effective detoxification, as it disrupts the planar conformation required for DNA intercalation and prevents metabolic activation by hepatic cytochrome P450 enzymes [45]. This structural detoxification was further evaluated by ProTox-3.0 simulations, which predicted a substantial loss of mutagenicity for the terminal decarboxylated metabolite (P2–P4).

As the biochemical transformation of aflatoxins is a complex metabolic process that rarely occurs via a single isolated enzyme in bacterial isolates, there were uniquely present enzymes in active degraders that were selected for further structural studies. In silico molecular docking simulations identified four promising candidate enzymes that exhibit high-affinity docking configurations with aflatoxins. Among these, paaH—a 3-hydroxybutyryl-CoA dehydrogenase involved in the phenylacetate degradation module (M00878) and mhpB demonstrated the most favorable binding affinities. Based on the fact that the four aflatoxins share a highly conserved, rigid tetracyclic/pentacyclic structure containing a difuran ring system fused to a coumarin nucleus, the key sites for an enzymatic attack are the difuran ring systems and the lactone ring [40]. Concerning paaH, paaH is an oxidoreductase enzyme that acts on the CH-OH group of donors utilizing NAD+ or NADP+ as hydrogen acceptors. Structurally, the docking model highlights a specialized substrate-anchoring mechanism within the catalytic pocket, mediated by residues ALA69, ALA70, and ASN71. The small, hydrophobic side chains of ALA69 and ALA70 form a tight hydrophobic surface. In the paaH native reaction, this region accommodates the fatty acyl-CoA chain; here, it provides crucial Van der Waals stabilization to the hydrophobic ring system of aflatoxins. Concurrently, the polar side chain of ASN71 participates in directional hydrogen bonding with the oxygen-rich lactone or ether groups of the toxin. This combination of hydrophobic shielding by ALA69/ALA70 and electrostatic anchoring by ASN71 suggests positioning the toxin within the catalytic pocket. In addition to the interaction profile of aflatoxin B2 with the catalytic enzyme mhpB. The complex is mainly stabilized by hydrophobic interactions involving VAL141, ALA143, VAL183, ALA187, LEU145, and LYS267, indicating that non-polar contacts dominate ligand accommodation within the active site. The present findings fit well with the broader literature on microbial aflatoxin degradation [46]. Hydrolases, dioxygenases, and oxidoreductase-related enzymes have repeatedly emerged as key players in aflatoxin catabolism [47,48]. Previous docking studies with recombinant laccases and F420H_2_-dependent reductases similarly identified strong spatial fit poses driven by hydrogen-bond networks and hydrophobic packing, with scores in the −6.4 to −5.5 kcal/mol range correlating with measurable degradation [49,50,51]. While molecular docking suggests high-affinity configurations, future work will include molecular dynamics (MD) simulations to fully account for protein flexibility and solvent effects.

This study integrates genomic reconstructions, LC-HR-MS/MS metabolite profiling, and molecular docking to provide a comprehensive, multi-scale blueprint of bacterial aflatoxin degradation. While in silico and genomic models point to candidate enzymes within active degrader catabolic pathways exclusively, experimental validation and heterologous expression remain essential. This step will help transition these isolated environmental strains into safe, scalable, and highly efficient enzymatic tools for food and feed decontamination.

## 5. Conclusions

In conclusion, this study addressed a critical bottleneck in aflatoxin mitigation by bridging the gap between classical physiological screening and high-throughput genomic resolution of aflatoxin-degrading bacteria. By combining Oxford Nanopore long-read sequencing, UPLC-HR-MS/MS mass shift profiling, and molecular docking, we have bypassed the limitations of fragmented marker-gene databases and unresolved biochemical pathways to deliver both biological resources and predictive structural insights. This study contributed to the deposition of six complete bacterial genomes into public databases, establishing a clear comparative genomic foundation that differentiates phenotypically active aflatoxin degraders from non-degraders. Active degraders demonstrated exceptional biological mitigation capabilities with 86–94% removal of aflatoxins B1, B2, G1, and G2. With *silico* toxicity modeling suggesting that the predicted demethylated and decarboxylated metabolites possess lower mutagenic potential than the parent toxin.

The presence of transmissible antimicrobial resistance (AMR) genes within these isolates shifts the application paradigm, establishing these strains as highly valuable, non-viable, enzyme-rich reservoirs for targeted bio-mining. By mining KEGG pathway profiles, we identified candidate oxidoreductases, hydrolases, and dioxygenases exclusively present in the active degrader strains. Molecular docking simulations resolved the enzyme-ligand compatibility structures, yielding exceptionally high docking scores that indicate strong binding affinity with aflatoxins. Thus, the present study has laid the groundwork to transition these predictive, structurally compatible candidate enzymes into heterologously expressed, safe, and highly purified biocatalysts. Future work will focus on the quantitative expression, purification, and wet-lab validation of these prioritized enzymes to unlock their full potential if they are safe and highly effective biological agents for industrial aflatoxin decontamination.

## Figures and Tables

**Table 1:** Comparative efficacy of viable bacteria, cell-free supernatant (CFS), and heat-killed biomass (HKC) on total aflatoxin removal mean (B1, G1, B2, G2) across the four active environmental isolates.

Strain	Source	Mean (%) for Viable Bacteria	Mean (%) for CFS	Mean (%) for HKC
2	Raw Milk	86.57 ± 4.17	73.40 ± 0.15	16.62 ± 1.72
4	Fecal	93.83 ± 0.14	79.13 ± 1.35	12.58 ± 2.34
5	Soil	94.02 ± 0.35	71.04 ± 2.11	9.14 ± 3.68
6	Raw Milk	90.90 ± 3.66	81.31 ± 1.52	12.13 ± 2.98

Values represent the mean % of total aflatoxin removal ± standard deviation (SD) of triplicate experiments (*n* = 3).

**Table 2:** Two-way ANOVA evaluating the effects of microbial strain and treatment preparation type on aflatoxin removal.

Source	*p*-Value	Partial η^2^
Strain	0.149	0.711
Type	0.001 *	0.952
Strain × Type	0.178	0.672

* Statistically significant at *p* < 0.05.

**Table 3:** Time-course analysis of aflatoxin removal efficiency (%) by Isolate 5.

Time (h)	OD 600	AFG1	AFB1	AFG2	AFB2
0	0.12	0	0	0	0
6	0.15	12.45 ± 2.85	18.3 ± 1.12	8.15 ± 0.95	6.2 ± 0.78
12	0.24	45.6 ± 1.42	52.18 ± 1.65	31.4 ± 1.55	24.5 ± 1.32
18	0.32	82.15 ± 1.15	88.45 ± 0.98	65.8 ± 1.88	54.12 ± 1.62
24	0.38	97.88 ± 2.32	99.43 ± 0.15	84.67 ± 1.20	74.33 ± 1.45
48	1.24	99.63 ± 0.11	99.75 ± 2.08	88.17 ± 0.65	81.83 ± 0.95
72	1.35	99.70 ± 1.06	99.83 ± 1.04	89.25 ± 0.45	92.58 ± 0.62

Values represent the mean % of total aflatoxin removal ± standard deviation (SD) of triplicate experiments.

**Table 4:** Morphological and biochemical validation profiles of environmental isolates according to Bergey’s Manual of Systematic Bacteriology.

Test/Isolate	Gram Stain	Catalase	Simmons Citrate	Urease	Triple Sugar Iron (TSI)	Kovac’s (Indole)	MacConkey	EMB	Result
1	Pink/red Rod-shaped (bacilli)	+	−	− Yellow slant	Yellow slant & Butt Gas Production No H_2_S	+	Growth, pink/pale	Metallic green sheen	*Escherichia coli*
2	Pink/red Rod-shaped (bacilli)	+	−	− Yellow slant	Yellow slant & Butt Gas Production No H_2_S	+	Growth, Bright pink	Metallic green sheen	*Escherichia coli*
3	Pink/red Rod-shaped (bacilli)	+	−	− Yellow slant	Yellow slant & Butt Gas Production No H_2_S	+	Growth, Bright pink	Metallic green sheen	*Escherichia coli*
4	Pink/red Rod-shaped (bacilli)	+	+	+ Pink slant	Yellow slant & Butt Gas Production No H_2_S	−	Growth, pink/pale	Dark pink, mucoid colonies	*Klebsiella pneumoniae*
5	Pink/red Rod-shaped (bacilli)	+	+	+ Pink slant	Yellow slant & Butt Gas Production No H_2_S	−	Growth, pink/pale	Dark pink, mucoid colonies	*Klebsiella pneumoniae*
6	Pink/red Rod-shaped (bacilli)	+	+	+ Pink slant	Yellow slant & Butt Gas Production No H_2_S	−	Growth, pink/pale	Dark pink, mucoid colonies	*Klebsiella pneumoniae*

**Table 5:** Putative treatment-associated biotransformation products detected in the CFS of *E. coli* EC_2 and *K. pneumoniae* KP_6.

Product ID	*E. coli* EC_2 (2E) Observed Precursor (*m*/*z*), Retention Time	*Klebsiella* KP_6 (6E) Observed Precursor (*m*/*z*), Retention Time	Mass Adduct	Peak Area (Standard Control)	Theoretical Precursor (*m*/*z*)	Putative Reaction Pathway
P1	*m*/*z* 299.0558, RT 6.892	*m*/*z* 299.0552, RT 6.886	[M+H]^+^	ND	299.0551	Demethylation
P2	*m*/*z* 269.0815, RT 9.123	*m*/*z* 269.0811, RT 9.117	[M+H]^+^	ND	269.0809	Decarboxylation
P3	*m*/*z* 269.0873, RT 10.953	*m*/*z* 269.0871, RT 10.936	[M+H]^+^	ND	269.0809	Decarboxylation
P4	*m*/*z* 269.0818, RT 14.671	*m*/*z* 269.0814, RT 14.662	[M+H]^+^	ND	269.0809	Decarboxylation

ND: Not Detected.

**Table 6:** Quick Vina 2 binding affinity scores (kcal/mol) of key xenobiotic enzymes against aflatoxin congeners.

Enzyme Candidate	AFG1	AFG2	AFB1	AFB2	Enzymatic Class/Role
paaH	−8.0	−8.7	−8.4	−8.3	Oxidoreductases K00074
mhpB	−6.0	−6.4	−6.1	−6.7	Dioxygenases in aromatic ring cleavage K05713
benA-xylX	−6.3	−5.8	−5.8	−6.3	Dioxygenases in aromatic ring cleavage K05549
pcaD	−5.8	−5.4	−5.6	−5.6	Hydrolases K01055
mhpE	+25.4	27.1	−0.43	−0.8	Negative control from EC_1 K01666

## Data Availability

The data presented in this study are openly available in NCBI at https://www.ncbi.nlm.nih.gov/bioproject/PRJNA1453922 (accessed on 1 September 2026), reference number PRJNA1453922.

## Supplementary Materials

The following supporting information can be downloaded at: https://www.mdpi.com/article/10.3390/foods15183242/s1, Figure S1: UV–visible spectra illustrating coumarin tolerance by bacterial isolates (2, 4, 5, and 6). Bacterial treated groups show significant reduction. Figure S2. Biochemical identification according to Bergey’s manual showing the bacterial species belonging to Enterobacteriaceae, (a) identifies strain 2: *E. coli*, (b–d) are isolates (4–6) characterized as *Klebsiella* sp. Table S1: sequencing, assembly, and annotation results; Table S2: representative protein-ligand interactions.

## Abbreviations

SDG	Sustainable Development Goals
ANOVA	Two-way analysis of variance
HPLC	High-Performance Liquid Chromatography
DMSO	Dimethyl Sulfoxide
ATCC	American Type Culture Collection
CFU/mL	Colony-Forming Units per Milliliter
SD	Standard Deviation
BLAST	Basic Local Alignment Search Tool
AMR	Antimicrobial Resistance
CARD	Comprehensive Antibiotic Resistance Database
NCBI	National Center for Biotechnology Information
MMFF94	Merck Molecular Force Field 1994
PDBQT	Protein Data Bank, Partial Charge (Q) & Atom Type (T) format
WGS	Whole Genome Sequencing
CB-Dock2	Cavity-Based Docking Tool version 2
SEM	Standard Error of the Mean
UPLC-HR-MS/MS	Ultra-Performance Liquid Chromatography coupled with High-Resolution Tandem Mass Spectrometry
Q-TOF	Quadrupole-Time-of-Flight
