# Supplementary material for: Biodegradation of Aflatoxins by Four Novel Environmental Bacterial Isolates: A Comparative Genomic and Molecular Docking-Proof-of-Concept Study
Source: Foods. 2026 Sep 14;15(18):3242. doi: 10.3390/foods15183242 (PMC13606510; doi:10.3390/foods15183242)
Supplement: Supplementary file 1 [file foods-15-03242-s001.zip › Supplementary Figures S1 and S2.pdf]

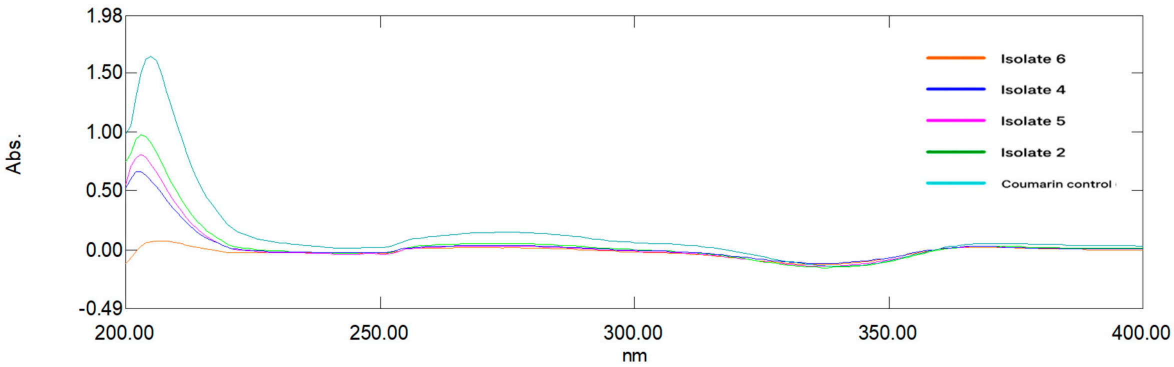

Figure S1. UV–visible spectra illustrating coumarin tolerance by bacterial isolates (2, 4, 5, and 6). Bacterial treated groups show significant reduction.

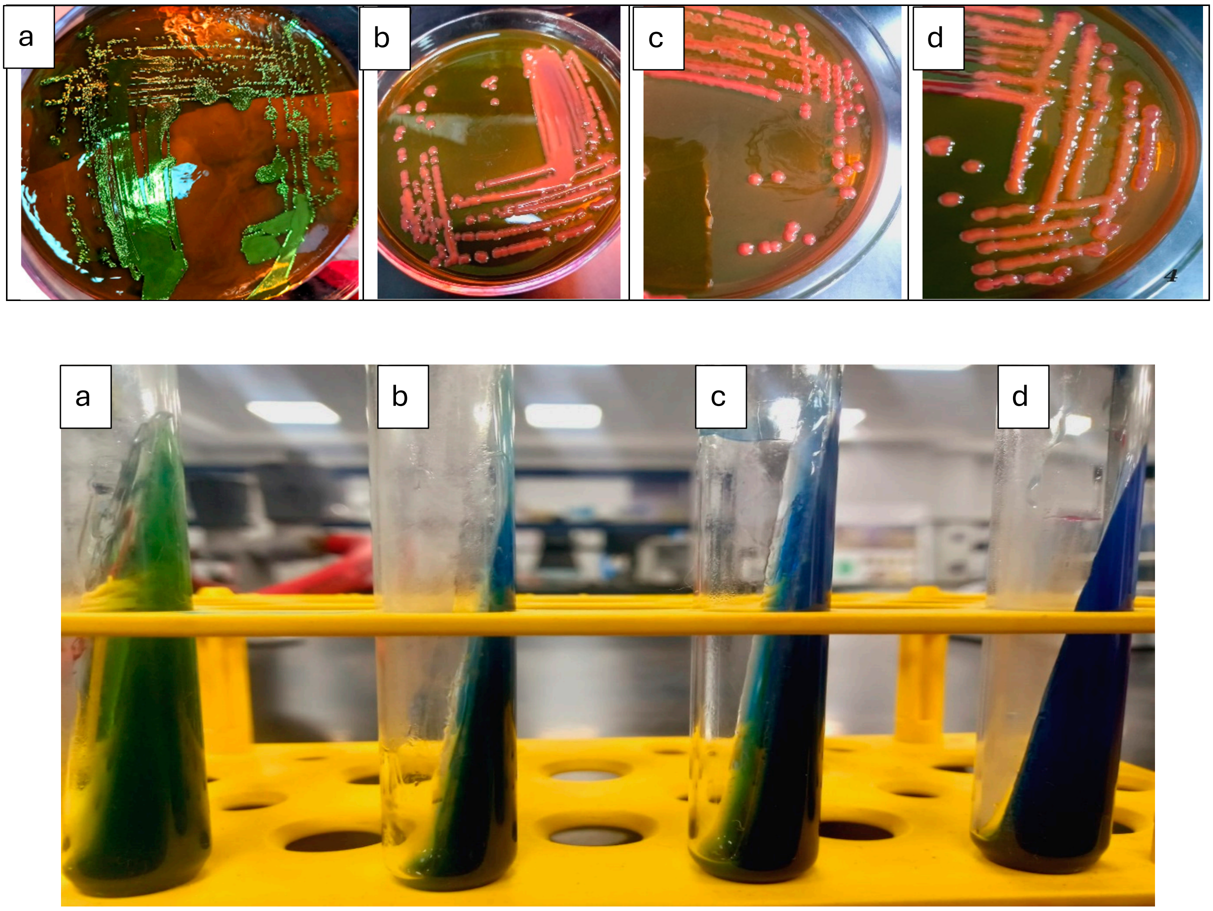

Figure S2: Biochemical identification according to Bergey's manual showing the bacterial species belonging to *Enterobacteriaceae,* (a) identifies strain 2: *E. coli*, (b-d) are isolates (4-6) characterized as *Klebsiella sp*.
